# Massive Acquired Renal Cysts Presenting with Bowel Obstruction-Like Symptoms

**DOI:** 10.1155/2022/5252051

**Published:** 2022-08-10

**Authors:** Chutikarn Teparak, Weeratian Tawanwongsri

**Affiliations:** Walailak University Hospital, 222 Taiburi, Thasala, Nakhon Si Thammarat 80161, Thailand

## Abstract

Acquired cystic kidney disease (ACKD) is rarely massive in size. The great majority is asymptomatic and incidentally found on imaging studies for unrelated causes. We reported a case of an 85-year-old male with bilateral multiple huge acquired renal infected cysts, initially presenting with bowel obstruction-like symptoms. The computed tomography (CT) scan later aided in an accurate diagnosis. Symptomatic huge ACKD has, to our knowledge, scarcely been described. In addition, retroperitoneal lesions resulting in abdominal pain remain unusual and underrecognized in general practice.

## 1. Introduction

A cystic kidney disease is a group of conditions that cause fluid-filled sacs to form in or around the kidneys [1]. It is attributed to a broad spectrum of etiologies, including inherited, acquired, and systemic disease-related conditions. One of the most common causes is acquired cystic kidney disease (ACKD) [2, 3]. Whilst the great majority (approximately 86% of cases) are asymptomatic, certain patients may develop hematuria, lower back pain, and urinary tract infection [4, 5]. Abdominal pain is thus claimed to be an unusual presentation. Various pain characteristics have been described, including dull, uncomfortable fullness, stabbing, and cramping [6]. We herein reported a rare case of an elderly patient who was ultimately diagnosed with infected ACKD, initially presented with acute abdominal pain.

## 2. Case Presentation

**Figure 1:**
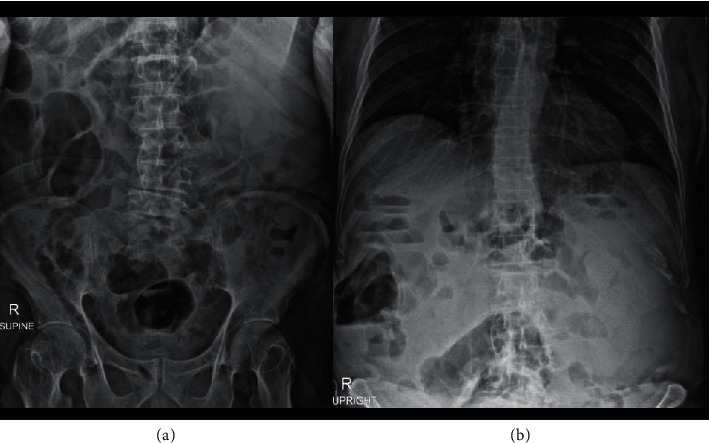
Supine (a) and upright (b) abdominal radiographs revealed a large well-defined soft tissue opacity at the left upper quadrant of abdomen, deviating left-sided colon medially. No signs of bowel obstruction were noted.

**Figure 2:**
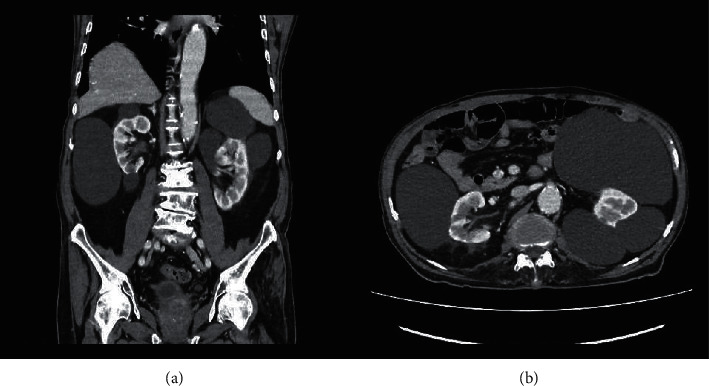
Coronal (a) and axial (b) CT images of the abdomen revealed multiple, variable-sized, thin-walled cysts in both kidneys. The diameter of the cysts ranged from 3.5–13.0 cm. Some renal cysts had higher attenuation with perinephric fat stranding, representing infected renal cysts.

An 85-year-old male was evaluated at this hospital because of acute abdominal pain. His comorbidities included essential hypertension, old myocardial infarction, chronic kidney disease, and gouty arthritis. No family history of early-onset chronic kidney disease was documented. Two years earlier, he had the first episode of urinary tract infection (UTI), which was resolved with a ten-day course of oral antibiotics at another hospital. One week after treatment, he underwent a kidney ultrasound examination to evaluate the possibility of complicated UTI. However, it revealed no abnormalities. The patient had been in his usual state of health until ten days before admission to our hospital, when acute abdominal pain, constipation, early satiety, and nausea developed. Abdominal pain was generalized, dull, 6 out of 10 in severity, and gradually worsened. He denied other organ-specific symptoms, except for a low-grade fever. He had never undergone abdominal surgery. On examination, the temperature was 38.4°C, the blood pressure was 127/73 mm·Hg, the heart rate was 86 beats per minute, and the respiratory rate was 18 breaths per minute. His abdomen was soft and moderately distended with slight but diffuse tenderness to soft touch and normal bowel sounds. The remainder of the examination was normal. A digital rectal examination revealed hard, brown stool with no blood or mucus. Plain film of the abdomen demonstrated a large well-defined high-density shadow on the left upper quadrant abdomen, deviating left colon to the central abdomen, without significant bowel dilatation (Figure 1). He then underwent computed tomography (CT) scan of the abdomen, which disclosed multiple, variable-sized, thin-walled, well-marginated cysts in both kidneys (Figure 2). The diameter of the cysts ranged from 3.5 to 13.0 cm, and it showed internal hyperdensity (16–20 Hounsfield units). Both kidney sizes were of normal size and had normal parenchyma without solid components. A complete blood count revealed a leukocyte count of 12,000 cells/mm^3^ with neutrophil predominance (79%). His serum creatinine was 2.13 mg/dL without proteinuria. Urinary analysis showed leukocyturia (a urine white blood cell count of 30–50 cells/high power field) with nitrite positive. After he was diagnosed with bilateral multiple infected acquired renal cysts, ciprofloxacin was administered intravenously. Four days later, his fever and abdominal pain had largely diminished. The hemoculture was reported negative and urine culture grew *Escherichia coli*. After his clinical conditions improved, ciprofloxacin was switched to oral form, and he was referred to a urologist for further evaluation and considering an evaluation for kidney transplantation.

## 3. Discussion

A spectrum of adult renal cystic diseases was categorized into hereditary and non-hereditary conditions. The common causes in patients with multiple renal cysts remain autosomal dominant polycystic kidney disease (ADPKD), ACKD, and simple renal cysts [2, 3]. Our patient was diagnosed with acquired renal cysts since his kidney sizes were of normal size and he had normal parenchyma. Yet, there were neither extrarenal cysts nor a positive family history of early-onset chronic renal diseases nor other organ-specific manifestations. His impaired renal function made the diagnosis of simple renal cysts unlikely [7, 8]. In addition, the congenital causes are less likely attributed to his clinical presentations because of his old age and no abnormal findings on the previous kidney ultrasound.

The four cardinal symptoms of bowel obstruction are pain, vomiting, obstipation (or constipation), and distention [9]. However, these tetrads were neither specific (28–61%) nor sensitive (56–75%) [10]. Therefore, the clinical features alone might not aid in recognizing and promptly managing bowel obstruction. Our patient presented with bowel obstruction-like symptoms with no history of abdominal surgery. Aside from partial bowel obstruction, conditions that had to be considered in a differential diagnosis included acute colonic pseudo-obstruction, mesenteric ischemia, and mesenteric artery ischemia [11]. A plain film of the abdomen revealed a mass-like shadow on the left upper quadrant abdomen without any evidence of bowel dilatation. Furthermore, CT abdomen revealed bilateral multiple huge renal cysts extended anteriorly toward intra-abdominal organs. The stretched peritoneum reasonably explains his generalized abdominal pain [12]. This case reminds us that a pattern recognition process or an intuitive approach in diagnosis has its limits in certain situations, particularly with highly incoherent data. Alternative strategies for generating differential diagnosis include anatomic approach, and systems approach, under a hypothetico-deductive method with critical thinking and clinical reasoning, may provide a more comprehensive range of possible conditions in this clinical setting [13].

ACKD is a condition commonly found in patients with advanced chronic kidney disease, presenting with multiple bilateral cysts, which are usually between 0.5 and 3 cm in diameter [4]. The prevalence of ACKD was approximately 7% and 22% in pre-dialysis patients and dialysis patients, respectively [14]. Common imaging techniques including ultrasonography, CT scan, magnetic resonance imaging (MRI) play an indispensable role in the diagnosis, prognostic staging, and management. In spite of the fact that ultrasound provides the most cost-effective option, CT and MRI demonstrate greater resolution with a high sensitivity and specificity particularly for detecting renal cysts less than one centimeter in diameter [15, 16]. Whilst its pathogenesis had not been well understood, it was postulated that nephron loss contributed to compensatory kidney hypertrophy, resulting in tubular hyperplasia and cyst formation. The process was mediated by activation of proto-oncogenes—which was presumably attributed to renal cell carcinoma (RCC)—and release of growth factors [17, 18]. The cyst stems from proximal tubules, and its fluid derives from the ultrafiltrate, transepithelial solute, and fluid secretion [19, 20]. The incidence of RCC was 4–7% over 7–10 years of follow-up [21,22]. To date, there is no consensus regarding the proper modality and frequency of imaging to detect RCC progression from ACKD. However, modalities—reported in previous studies—included ultrasound, CT, and magnetic resonance imaging (MRI) [21, 23–25]. Among imaging studies for diagnosing renal cell carcinoma, the diagnostic performance of unenhanced ultrasound was poor. Vogel et al. revealed that median sensitivity and specificity of CT abdomen were 88% and 75%, respectively. Also, median sensitivity and specificity of MRI abdomen were 87.5% and 89%, respectively [26].

According to his cyst infection, common pathogens included *Escherichia coli, Klebsiella pneumoniae, Salmonella enteritidis, Citrobacter diversus, Enterococcus faecalis,* and *Enterococcus* spp. [27]. Lipid-soluble antibiotics—for instance, fluoroquinolones—demonstrated excellent penetration into cysts; however, resistant strains were increasingly being identified [27, 28]. Because of their properties, these regimens were favored [29]. The ACKD patients with pain or abdominal fullness from mass effect are treated symptomatically. Because of the rarity of cases with symptoms, the gold standard of definite treatment in patients with ACKD has not been well established. Based on previous studies of autosomal dominant polycystic kidney disease, nephrectomy may be needed in cases with intractable pain and cyst-related complications including retroperitoneal bleeding, persistent or severe hematuria, recurrent and severe cyst infection, infected stones, and suspicion of renal cell carcinoma [30]. Moreover, a recent study revealed that trans-arterial embolization of enlarged polycystic kidneys appears to be an advantageous alternative to nephrectomy before renal transplantation [31]. In patients with kidney transplants, the recipient kidney was smaller in size and had milder grade of severity compared to those treated with hemodialysis [32]. Although no recurrence of his symptoms had been detected, he was referred to experienced nephrologists and urologists for an early discussion of the surgical intervention. After discussion, the preferred management was close monitoring and the invasive intervention would be re-considered if indicated. In our case, no evidence of RCC had yet been found in CT abdomen findings. We advised our patient to undergo CT abdomen scanning to assess the progression of the disease and screen for RCC every 1-2 years, based on its growth rate of 1 cm per year [33, 34].

## 4. Conclusion

Our case exemplifies the importance of constructing a wide differential diagnosis in patients without prior abdominal surgery presenting with bowel obstruction-like symptoms. Retroperitoneal lesions, although rare, can cause abdominal pain and bowel obstruction-like symptoms. Careful radiographic examination and thoughtful evaluation of clinically relevant information is of importance to establish an accurate diagnosis and provide proper treatment.

## Data Availability

All data that support the findings of this study are included in this article. Further enquiries can be directed to the corresponding author.

## References

[1] Kurschat C. E., Müller R. U., Franke M., Maintz D., Schermer B., Benzing T. (2014). An approach to cystic kidney diseases: the clinician’s view. *Nature Reviews Nephrology*.

[2] Alves M., Fonseca T., de Almeida E. A. (2015). *Differential Diagnosis of Autosomal Dominant Polycystic Kidney Disease*.

[3] Katabathina V. S., Vinu-Nair S., Gangadhar K., Prasad S. R. (2015). Update on adult renal cystic diseases. *Applied Radiology*.

[4] Rahbari-Oskoui F., O’Neill W. C. (2017). Diagnosis and management of acquired cystic kidney disease and renal tumors in ESRD patients. *Seminars in Dialysis*.

[5] Truong L. D., Krishnan B., Cao J. T., Barrios R., Suki W. N. (1995). Renal neoplasm in acquired cystic kidney disease. *American Journal of Kidney Diseases*.

[6] Bajwa Z. H., Sial K. A., Malik A. B., Steinman T. I. (2004). Pain patterns in patients with polycystic kidney disease. *Kidney International*.

[7] Nahm A. M., Ritz E. (2001). Acquired renal cysts. *Nephrology Dialysis Transplantation*.

[8] Pei Y. (2010). Practical genetics for autosomal dominant polycystic kidney disease. *Nephron Clinical Practice*.

[9] Kulaylat M. N., Doerr R. J. (2001). Small bowel obstruction: NCBI bookshelf. https://www.ncbi.nlm.nih.gov/books/NBK6873.

[10] Al Salamah S. M., Fahim F., Hameed A. M. A., Abdulkarim A. A., Al Mogbal E. S., Al Shaer A. (2012). How predictive are the signs and symptoms of small bowel obstruction. *Oman Medical Journal*.

[11] Jackson P., Vigiola Cruz M. (2018). Intestinal obstruction: evaluation and management. *American Family Physician*.

[12] Struller F., Weinreich F.-J., Horvath P. (2017). Peritoneal innervation: embryology and functional anatomy. *Pleura and peritoneum*.

[13] Croskerry P. (2009). A universal model of diagnostic reasoning. *Academic Medicine*.

[14] Narasimhan N., Golper T. A., Wolfson M., Rahatzad M., Bennett W. M. (1986). Clinical characteristics and diagnostic considerations in acquired renal cystic disease. *Kidney International*.

[15] Chapman A. B., Wei W. (2011). Imaging approaches to patients with polycystic kidney disease. *Seminars in Nephrology*.

[16] Pei Y., Hwang Y. H., Conklin J. (2015). Imaging-based diagnosis of autosomal dominant polycystic kidney disease. *Journal of the American Society of Nephrology*.

[17] Konda R., Sato H., Hatafuku F., Nozawa T., Ioritani N., Fujioka T. (2004). Expression of hepatocyte growth factor and its receptor C-met in acquired renal cystic disease associated with renal cell carcinoma. *The Journal of Urology*.

[18] Oya M., Mikami S., Mizuno R., Marumo K., Mukai M., Murai M. (2005). C-jun activation in acquired cystic kidney disease and renal cell carcinoma. *The Journal of Urology*.

[19] Grantham J. J. (1991). Acquired cystic kidney disease. *Kidney International*.

[20] Grantham J. J., Levine E. (1985). Acquired cystic disease: replacing one kidney disease with another. *Kidney International*.

[21] Levine E., Slusher S. L., Grantham J. J., Wetzel L. H. (1991). Natural history of acquired renal cystic disease in dialysis patients: a prospective longitudinal CT study. *American Journal of Roentgenology*.

[22] Ishikawa I., Saito Y., Shikura N., Kitada H., Shinoda A., Suzuki S. (1990). Ten-year prospective study on the development of renal cell carcinoma in dialysis patients. *American Journal of Kidney Diseases*.

[23] Edo H., Suyama Y., Sugiura H. (2020). Acquired cystic disease-associated renal cell carcinoma extending to the renal pelvis mimicking urothelial carcinoma on computed tomography (CT): two case reports. *The American journal of case reports*.

[24] Marple J. T., MacDougall M., Chonko A. M. (1994). Renal cancer complicating acquired cystic kidney disease. *Journal of the American Society of Nephrology*.

[25] Matson M. A., Cohen E. P. (1990). Acquired cystic kidney disease: occurrence, prevalence, and renal cancers. *Medicine (Baltimore)*.

[26] Vogel C., Ziegelmüller B., Ljungberg B. (2019). Imaging in suspected renal-cell carcinoma: systematic review. *Clinical Genitourinary Cancer*.

[27] Suwabe T., Araoka H., Ubara Y. (2015). Cyst infection in autosomal dominant polycystic kidney disease: causative microorganisms and susceptibility to lipid-soluble antibiotics. *European Journal of Clinical Microbiology & Infectious Diseases*.

[28] Hamanoue S., Suwabe T., Ubara Y. (2018). Cyst infection in autosomal dominant polycystic kidney disease: penetration of meropenem into infected cysts. *BMC Nephrology*.

[29] Lantinga M. A., Casteleijn N. F., Geudens A. (2017). Management of renal cyst infection in patients with autosomal dominant polycystic kidney disease: a systematic review. *Nephrology Dialysis Transplantation: Official Publication of the European Dialysis and Transplant Association - European Renal Association*.

[30] Cornec-Le Gall E., Alam A., Perrone R. D. (2019). Autosomal dominant polycystic kidney disease. *The Lancet*.

[31] Cornelis F., Couzi L., Le Bras Y. (2010). Embolization of polycystic kidneys as an alternative to nephrectomy before renal transplantation: a pilot study. *American Journal of Transplantation*.

[32] Lien Y. H. H., Hunt K. R., Siskind M. S., Zukoski C. (1993). Association of cyclosporin A with acquired cystic kidney disease of the native kidneys in renal transplant recipient. *Kidney International*.

[33] Doublet J. D., Peraldi M. N., Gattegno B., Thibault P., Sraer J. D. (1997). Renal cell carcinoma of native kidneys: prospective study of 129 renal transplant patients. *The Journal of Urology*.

[34] Klatte T., Marberger M. (2011). Renal cell carcinoma of native kidneys in renal transplant patients. *Current Opinion in Urology*.

